# Single nucleotide polymorphism in the genes of *mce1 *and *mce4 *operons of *Mycobacterium tuberculosis*: analysis of clinical isolates and standard reference strains

**DOI:** 10.1186/1471-2180-11-41

**Published:** 2011-02-23

**Authors:** Rashmi Pasricha, Amita Chandolia, Prija Ponnan, Neeraj Kumar Saini, Sangeeta Sharma, Madhu Chopra, Mandira Varma Basil, Vani Brahmachari, Mridula Bose

**Affiliations:** 1 Department of Microbiology, Vallabhbhai Patel Chest Institute, University of Delhi, Delhi-110007, India; 2 Department of Biochemistry, Vallabhbhai Patel Chest Institute, University of Delhi, Delhi-110007, India; 3 The Centre for Genomic Application, Okhla Industrial Area, Delhi-110020, India; 4 Dr B.R. Ambedkar Center for Biomedical Research, University of Delhi, Delhi-110007, India

## Abstract

**Background:**

The presence of four mammalian cell entry (*mce*) operons in *Mycobacterium tuberculosis *suggests the essentiality of the functions of the genes in these operons. The differential expression of the four *mce *operons in different phases of *in vitro *growth and in infected animals reported earlier from our laboratory further justifies the apparent redundancy for these genes in the genome.

Here we investigate the extent of polymorphism in eight genes in the *mce1 *and *mce4 *operons of *M. tuberculosis *from four standard reference strains (H37Rv, H37Ra, LVS (Low Virulent Strain) and BCG) and 112 clinical isolates varying in their drug susceptibility profile, analysed by direct sequencing and Sequenom MassARRAY platform.

**Results:**

We discovered 20 single nucleotide polymorphisms (SNPs) in the two operons. The comparative analysis of the genes of *mce1 *and *mce4 *operons revealed that *yrbE1A *[*Rv0167*] was most polymorphic in *mce1 *operon while *yrbE4A *[*Rv3501c*] and *lprN *[*Rv3495c*] had the highest number of SNPs in the *mce4 *operon. Of 20 SNPs, 12 were found to be nonsynonymous and were further analysed for their pathological relevance to *M. tuberculosis *using web servers PolyPhen and PMut, which predicted five deleterious nonsynonymous SNPs. A mutation from proline to serine at position 359 of the native Mce1A protein was most deleterious as predicted by both PolyPhen and PMut servers. Energy minimization of the structure of native Mce1A protein and mutated protein was performed using InsightII. The mutated Mce1A protein showed structural changes that could account for the effects of this mutation.

**Conclusions:**

Our results show that SNPs in the coding sequences of *mce1 *and *mce4 *operons in clinical isolates can be significantly high. Moreover, *mce4 *operon is significantly more polymorphic than *mce1 *operon (p < 0.001). However, the frequency of nonsynonymous substitutions is higher in *mce1 *operon and synonymous substitutions are more in *mce4 *operon. *In silico *modeling predict that nonsynonymous SNP at *mce1A *[*Rv0169*], a virulence gene could play a pivotal role in causing functional changes in *M. tuberculosis *that may reflect upon the biology of the bacteria.

## Background

The variability in the genome sequence of *M. tuberculosis *between clinical isolates has been analysed earlier and variability in the number and site of integration of transposable element IS6110 is well documented [1]. There are also reports on the analysis of whole genome SNPs in mycobacteria [2]. Compared to many other bacterial species, *M. tuberculosis *exhibits very little genomic sequence variation [3]. However, there is increasing evidence that even this limited inter-strain genetic variability is biologically significant [4]. *M. tuberculosis *infection in animal models has shown a range of immune responses and variable degrees of virulence depending on the infecting strain [5,6]. In the majority of humans, an effective immune response develops after infection with *M. tuberculosis *and restricts the spread of the pathogen and clinical manifestation of the disease is seen in less than 10% of those infected. Clinical tuberculosis is influenced by variability in the host's genetic background, immune status, diet, social and environmental factors [7,8]. However, little is known about the bacterial factors, especially, genetic diversity in bacterial virulence factors contributing to variable host responses.

The expression of *mce *genes is of importance for the virulence of mycobacteria [9,10]. The presence of four copies of *mce *genes in four operons each consisting of eight genes [11] and the differential expression of *mce1 *and *mce4 *operons points towards functional importance of these operons [9,12]. Interestingly, the domain organization in the genes of all the four operons is similar. This conservative arrangement may be of strategic significance to the biology of *M. tuberculosis*. The antigenic and immunogenic effects of *mce *proteins in nature suggest that the variation in amino acid sequence of these proteins may affect host response, apart from their effect on functions of these proteins [13,14]. In the light of these observations, we initiated the present study to understand the possible importance of genetic diversity in the *mce *operon genes which have a role in the pathogenesis of *M. tuberculosis*. Polymorphism in the genes of *mce1 *and *mce4 *operons in 112 clinical isolates of *M. tuberculosis *was analysed to understand and relate the effect of the genetic variability to structural changes in the proteins by computational methods.

## Results

### Single nucleotide polymorphism in *mce *operons

**Figure 1:**
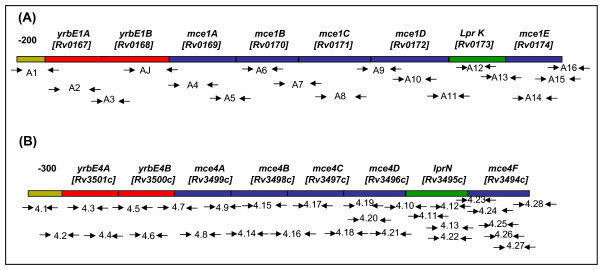
**Primers of *mce *operons**. Schematic representation of the position of overlapping primers to completely sequence the genes of (**A**) *mce1 *operon (**B**) *mce4 *operon.

We used a discovery platform consisting of four standard reference strains (H37Rv, H37Ra, LVS (Low Virulent Strain) and BCG) and 12 clinical isolates selected at random. Overlapping primers were designed to map eight genes each of *mce1 *and *mce4 *operons (Figure 1). We identified 7 SNPs in *mce1 *operon; 6 of these were nonsynonymous and one was synonymous substitution (Table 1). 100 clinical isolates were then genotyped for these SNPs on Sequenom MassARRAY platform. Among 8 genes of *mce1 *operon, *yrbE1A *[*Rv0167*] gene was significantly more polymorphic as compared to the other seven genes, with approximately 26% of clinical isolates showing the polymorphic allele. In comparison 13 SNPs were identified in *mce4 *operon (Table 2), of which 6 were nonsynonymous and 7 were synonymous SNPs. In *mce4 *operon significant polymorphism was observed in clinical isolates at *yrbE4A *[*Rv3501c*] and *lprN *[*Rv3495c*] genes with 25.50% and 26.50% SNP respectively.

**Table 1:** Polymorphisms in the genes of *mce1 *operon.

*mce1 *operon								
**Gene Name** **(Accession Number)**	**Nucleotide Change** **[GenBank Accession Number]**	**Amino Acid Change**		**Frequency Distribution of polymorphism** **(%)**				

		**Non Synonymous**	**Synonymous**	**All isolates** **n = 112**	**DS** **n = 22**	**DR** **n = 59**	**SDR** **n = 15**	**MDR TB** **n = 19**

*yrbE1A* [*Rv0167*]	C14T [GenBank:HQ901088]	Thr5Ile	NONE	(25.96)	(29.16)	(29.09)	(41.76)	(15.78)

*yrbE1B* *[Rv0168*]	T154G [GenBank:HQ901089]	Tyr52Asp	NONE	(0.9)	NONE	(1.72)	NONE	(5.26)

*mce1A* [*Rv0169*]	C1075T C1323T [GenBank:HQ901082]	Pr0359Ser	Tyr441Tyr	(1.87)	(4)	NONE	NONE	NONE

*mce1B* [*Rv0170*]	T536C [GenBank:HQ901085]	Ile179Thr	NONE	(0.9)	(3.8)	NONE	NONE	NONE

*mce1C* [*Rv0171*]	G636C [GenBank: HQ901086]	Glu212Asp	NONE	(0.9)	(3.8)	NONE	NONE	NONE

*mce1D* [*Rv0172*]	NONE	NONE	NONE	NONE	NONE	NONE	NONE	NONE

*lprK* [*Rv0173*]	NONE	NONE	NONE	NONE	NONE	NONE	NONE	NONE

*mce1F* [*Rv0174*]	G129T [GenBank: HQ901083]	Lys43Asn	NONE	(0.9)	(4)	NONE	NONE	NONE

Frequency of single nucleotide polymorphisms detected in the genes of *mce1 *operon. The nucleotide changes and the corresponding changes in amino acids are shown here. The frequency of SNPs was calculated from 112 clinical isolates. The data has been subdivided according to the drug susceptibility profile.

The single letter nucleotide designations used are as follows: A, adenine; C, cytosine; G, guanine and T, thymidine. The three letter amino acid designations used are as follows: Thr, threonine; Ile, isoleucine; Tyr, tyrosine; Asp, aspartic acid; Pro, proline; Ser, serine; Glu, glutamic acid; Lys, lysine and Asn, asparagine.

DS: drug sensitive, DR: drug resistant, SDR: single drug resistant, MDR TB: Multi drug resistant

**Table 2:** Polymorphisms in the genes of *mce4 *operon.

*mce4 *operon								
**Gene Name** **(Accession Number)**	**Nucleotide Change** **[GenBank Accession Number]**	**Amino Acid Change**		**Frequency Distribution of polymorphism** **(%)**				

		**Non Synonymous**	**Synonymous**	**All isolates** **n = 112**	**DS** **n = 22**	**DR** **n = 59**	**SDR** **n = 15**	**MDR TB** **n = 19**

*yrbE4A* [*Rv3501c*]	G18T C753A [GenBank: HQ901084]	NONE	Ala6Ala Ile251Ile	(25.49)	(20.83)	(29.62)	(41.76)	(21.05)

*yrbE4B* [*Rv3500c*]	C21T C624T [GenBank: HQ901090]	NONE	Ile7Ile Pro208Pro	(3.7)	(8)	(3.44)	(5.88)	NONE

*mce4A* [*Rv3499c*]	T32G C873T [GenBank: HQ901091]	Val11Gly	Phe291Phe	(2.25)	(4.55)	NONE	NONE	NONE

*mce4B* [*Rv3498c*]	NONE	NONE	NONE	NONE	NONE	NONE	NONE	NONE

*mce4C* [*Rv3497c*]	A136C C571A [GenBank: HQ901092]	Thr46Pro Arg191Ser	NONE	(3.75)	(8.33)	NONE	(5.88)	(5.26)

*mce4D* [*Rv3496c*]	G35A [GenBank: HQ901093]	Arg12Gln	NONE	(1.25)	(7.69)	NONE	NONE	NONE

*lprN* [*Rv3495c*]	C798T C1016A [GenBank: HQ901094]	Thr339Lys	Ala266Ala	(26.47)	(29.09)	(30.9)	(31.57)	(31.07)

*mce4F* [*Rv3494c*]	C117A C1214T [GenBank: HQ901087]	Pro405Lys	Thr39Thr	(8.75)	(9.09)	(7.3)	(10.52)	(5.09)

Frequency of single nucleotide polymorphisms detected in the genes of *mce4 *operon. The nucleotide changes and the corresponding changes in amino acids are shown here. The frequency of SNPs was calculated from 112 clinical isolates. The data has been subdivided according to the drug susceptibility profile.

The single letter nucleotide designations used are as follows: A, adenine; C, cytosine; G, guanine and T, thymidine. The three letter amino acid designations used are as follows Ala, alanine; Ile, isoleucine; Pro, proline; Val, valine; Gly, glycine; Phe, phenylalanine; Thr, threonine; Arg, arginine; Ser; serine; Gln, glutamine and Lys, lysine.

DS: drug sensitive, DR: drug resistant, SDR: single drug resistant, MDR TB: Multi drug resistant

### Effect of SNPs on codon usage in *mce *operons

The preferential usage of codons for different amino acids in various organisms including *M. tuberculosis *is well known. The codon bias influences the translational efficiency in these organisms [15]. Therefore, we analysed the codon usage in *M. tuberculosis *for synonymous changes observed in both *mce1 *and *mce4 *operons. Analysis revealed that codons of amino acids were changed to the next preferred codon (Table 3). It is possible that such altered preference for certain codons would alter the expression of the respective proteins.

**Table 3:** Codon usage in *mce1 *and *mce4 *operons

Operon	Gene name (Accession Number)	Wild type codon	Polymorphic codon
***mce1 *operon**	*mce1A* [*Rv0169*]	TAC	TA**T**

	*yrbE4A* [*Rv3501c*]	GCG ATC	GC**T** AT**A**
	
***mce4***	*yrbE4B* [*Rv3500c*]	ATC CCC	AT**T** CC**T**
	
**operon**	*mce4A* [*Rv3499c*]	TTC	TT**T**
	
	*lprN* [*Rv3495c*]	GCC	GC**T**
	
	*mce4F* [*Rv3494c*]	ACC	AC**A**

The codon usage in the polymorphic regions is shown here. The synonymous changes in the nucleotide sequence, when analysed bioinformatically through Gene Runner software version 3.05 (Hastings Software, Inc.) predicts the usage of less preferred codon which could reflect upon the expression efficiency of the protein encoded by the gene.

Nucleotide highlighted in bold indicates the altered nucleotide.

### Prediction of functional consequences of nonsynonymous SNPs by PolyPhen and PMut servers

The functional impact of 12 nonsynonymous SNPs in proteins of *mce1*and *mce4 *operons was analyzed using PolyPhen http://genetics.bwh.harvard.edu/pph/ and PMut http://mmb2.pcb.ub.es:8080/PMut/ servers. Of the 12 nonsynonymous SNPs studied, 5 nonsynonymous SNPs were predicted to be deleterious to the organism by both PolyPhen and PMut programs. These nonsynonymous SNPs were located in the genes *yrbE1B *[*Rv0168*] (NN output; 0.84, PSIC score; 1.6), *mce1A *[*Rv0169*] (NN output; 0.84, PSIC score; 2.04), *mce1B *[*Rv0170*] (NN output; 0.59, PSIC score; 1.6), *lprN *[*Rv3495c*] (NNoutput; 0.55, PSIC score; 1.73) and *mce4F *[*Rv3494c*] (NN output; 0.52, PSIC score; 2.01). Whereas the other 7 nonsynonymous SNPs had NN output < 0.5 and PSIC score < 1.5. The highest score in this analysis was for *mce1A *gene with C1075T mutation resulting in substitution of proline to serine at 359 amino acid position. Thus, C1075T was considered to be the most deleterious mutation by PolyPhen and PMut programs.

### Modeling of mutated protein structure

**Figure 2:**
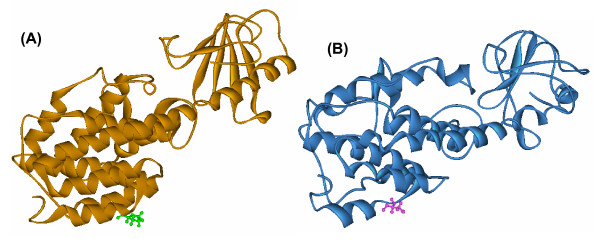
**Wild and mutant protein structure of Mce1A**. Structure of (**A**) wild (orange ribbon) and (**B**) Pro359Ser mutant (blue ribbon) proteins showing Pro359 (green) in wild protein and Ser359 (pink) in the mutant protein represented in ball and stick. The figure was prepared using Discovery studio 2.5 (DS Modeling 2.5, Accelrys Inc.: San Diego, CA).

**Figure 3:**
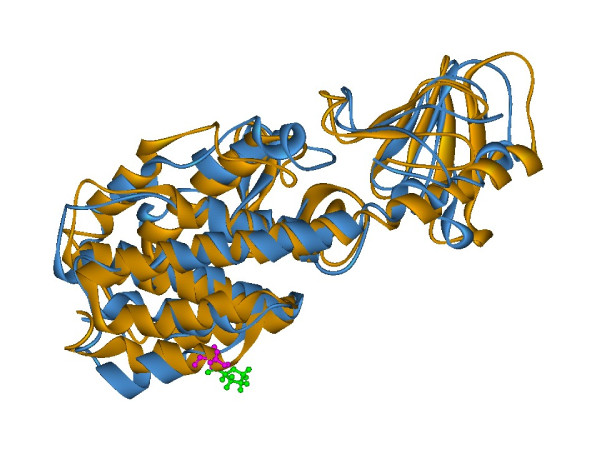
**Comparison of Wild and mutant protein structure of Mce1A**. Superimposed structure of wild (orange) and Pro359Ser mutant (blue) of Mce1A protein showing a change in helix to loop conformation after energy minimization of protein structures, as described in methods section. The RMSD between native and mutant protein was 3.07Ǻ. Pro359 (green) in wild protein and Ser359 (pink) in the mutant protein are represented in ball and stick.

**Figure 4:**
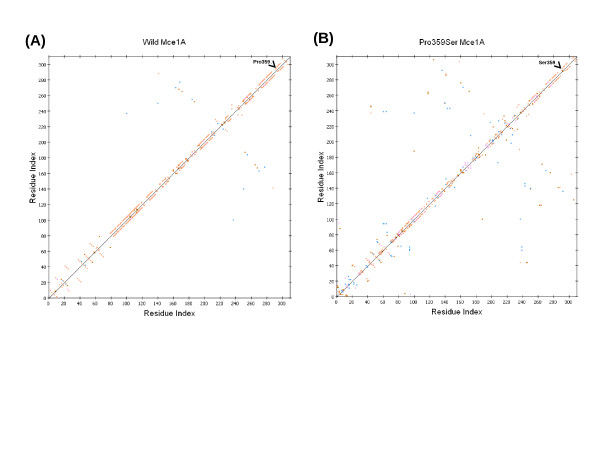
**HB plot representation of wild and mutant Mce1A protein**. HB plot of wild (**A**) and Pro359Ser mutant (**B**) Mce1A protein. Break in the diagonal at position 359 in the HB plot of Pro359Ser indicates loss of hydrogen bond after mutation. Conformational changes in other regions could be attributed to the alteration of hydrogen bonds in these regions. Colours of the dots in the HB plot indicated the type of hydrogen bond interactions: side chain-side chain (blue), main chain-main chain (orange), main chain-side chain (red) and multiple hydrogen bonds between amino acid residues (pink) The figures were prepared using Discovery studio 2.5 (DS Modeling 2.5, Accelrys Inc.: San Diego, CA).

We selected C1075T (Pro359Ser) polymorphism in *mce1A *gene as shown in Table 1 for further structural analysis. The substitution is positioned at 359 amino acid and we have mapped this in the three dimensional structure [PDB: 1NA9] [16]. Mutation at the specified position was performed by InsightII/Biopolymer and energy minimizations were performed by InsightII/Discover module for both the native structure [PDB: 1NA9] and mutant modeled structure (Pro359Ser). This structural analysis shows that the native (Figure 2A) and the mutant (Figure 2B) protein structure has an RMSD of 3.07 Ǻ. It is interesting to observe that, in the native structure, Proline359 is a part of the helical conformation while the mutated counterpart (Pro359Ser) has a loop structure at this position (Figure 3). Perturbation in the hydrogen bonds as indicated in the HB plots (Figure 4A and 4B) could be attributed to the conformational changes at Ser 359 position and other regions of mutant protein.

### Correlation between drug resistance and SNPs

**Figure 5:**
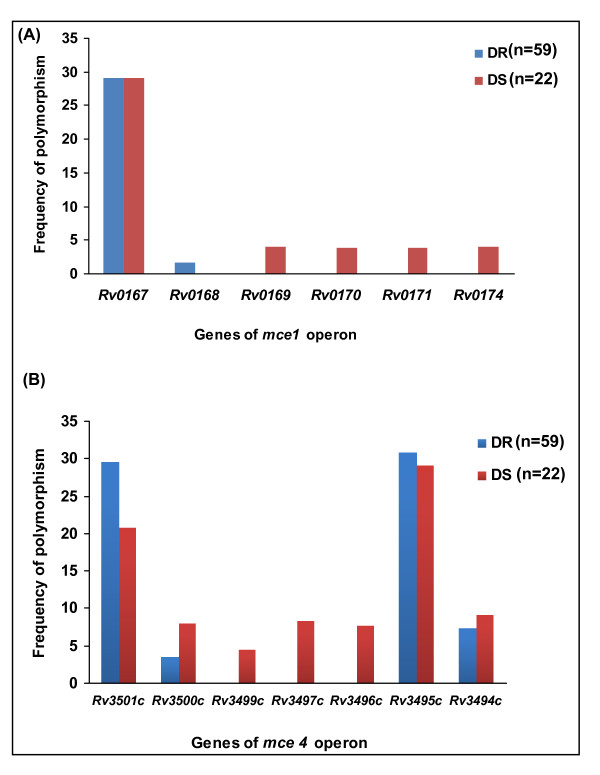
**Comparative analysis of the frequency of SNPs in the *mce *operons genes of drug resistant (DR) and drug sensitive (DS) clinical isolates**. SNPs were explored using Sequenom MassARRAY platform. DR (n = 59) and DS (n = 22) clinical isolates of *M. tuberculosis *were taken up for this study. The comparison between the two categories revealed that (**A**) *mce1 *and (**B**) *mce4 *operon genes were significantly more polymorphic in DS clinical isolates than DR isolates (*, p < 0.05) and (**, p < 0.01) respectively.

Approximately 59 drug resistant (DR) and 22 drug sensitive (DS) clinical isolates were analysed further. Four first line drugs namely isoniazid, rifampicin, ethambutol and streptomycin were taken into account to characterize the isolates. The sensitive isolates were sensitive to all the four antitubercular drugs while the resistant isolates were resistant to atleast one drug. The comparison between the two categories revealed that *mce1 *and *mce4 *operon genes were significantly more polymorphic in DS clinical isolates than DR isolates (*, p < 0.05) (Figure 5A) and (**, p < 0.01) (Figure 5B) respectively.

Among 59 DR clinical isolates, 19 were MDR TB (Multi drug resistant, at least to isoniazid and rifampicin). Among 19 MDR TB clinical isolates, polymorphism was observed in *yrbE1A *(15.78%) and *yrbE1B *(5.26%) genes of *mce1 *operon; and in *yrbE4A *(21.05%), *mce4B *(5.26%), *lprN *(31.57%) and *mce4F *(10.52%) genes of *mce4 o*peron. Of the 15 single drug resistant (SDR) clinical isolates studied, polymorphism was observed in *yrbE1A *(41.76%) gene of *mce1 *operon and in *yrbE4A *(41.76%), *yrbE4B *(5.88%), *mce4C *(5.26%), *lprN *(35.29%) and *mce4F *(5.88%) genes of *mce4 *operon. Interestingly, *mce *genes were significantly more polymorphic in SDR strains than MDR TB strains in both *mce1 *and *mce4 *operons. (**, p < 0.01 and ***, p < 0.001 respectively).

## Discussion

It has been observed that severity of tuberculosis varies in different patients. It is possible that clinical isolates of *M. tuberculosis *encountering the human hosts with individual immune systems need to accordingly modulate their virulence associated biological factors to survive within the host. Therefore, it is important to understand the biology of the pathogen at the genetic level. Genetic polymorphisms in the bacterial hosts have been shown to significantly influence the biology of the organisms [17]. In *M. tuberculosis*, most of the polymorphisms have been studied in the transposable elements and drug resistant genes [1,18]. A study of the genetic mutations in the genes coding for virulence factors interacting with host's immune system would help us in understanding the ways in which various strains of *M. tuberculosis *adapt to different hosts.

The sequencing and Sequenom MassARRAY analysis presented here have revealed that *mce4 *operon is significantly more polymorphic than *mce1 *operon. Seven out of eight genes of *mce4 *operon were found to be polymorphic. The *mce4 *operon is expressed in the stationary phase of *in vitro *broth culture and in the late phase of infection [9,12] and has role in the survival of the pathogen inside the host body [19]. This is the time when the bacterium has established itself efficiently in the host and it is possible that the bacterium then permits itself to undergo genetic substitutions to evade the host immune response. The detailed analysis of codon usage for synonymous changes observed in both *mce1 *and *mce4 *operons revealed that codons of amino acids were changed to the next preferred codon which would alter the expression of proteins. Our observation of more codon bias in *mce4 *operon that may lead to less expression of proteins further supports the possibility that such diversity facilitates better survival of *M. tuberculosis *inside the host's body. Our results further reveal that more than 25% of clinical isolates have SNPs in *yrbE4A *and *lprN *genes of *mce4 *operon. The *lprN *gene of *mce4 *operon codes for lipoprotein precursor [20]. The lipoproteins of *M. tuberculosis *are known to be effectively antigenic in nature [21]. Thus, high polymorphism in *lprN *gene (both synonymous and nonsynonymous) further supports our hypothesis that such polymorphisms favour intracellular survival of the pathogen. Drug resistance itself makes the organism in a better position to survive within the hostile intracellular environment. But DS isolates being drug susceptible do not have this advantage. Therefore antigenic variation is a tool utilized by DS clinical isolates. For example, the function of PPE proteins is unknown. However several observations and results support that many are cell surface associated and recognized by the host immune system. The possibility of high antigenic variation associated with these highly antigenic PE and the PPE family proteins have also been reported [22]. The PGRS member *Rv1759 *is a fibronectin-binding protein of relative molecular mass 55,000 Da [23] that elicits a variable antibody response, indicating either that individuals mount different immune responses or that this PGRS protein may vary between strains of *M. tuberculosis*. Bioinformatics analysis have indicated that LprN is also a cell surface associated protein. Therefore it is possible that SNP observed in this gene could be translated into antigenic variation in the LprN protein to facilitate the intracellular survival of mycobacteria.

In contrast, the *mce1 *operon is required for the entry of the pathogen inside the host cell [24] and hence, remains less polymorphic. However, the *yrbE1A *gene is revealed to be highly polymorphic in *mce1 *operon. Since, YrbE1A has been predicted to be a transmembrane protein [20], so the observed polymorphism in its gene may influence activity of the protein.

From the computational analysis, we could infer that the results obtained on the basis of structural details (PolyPhen) and sequence details (PMut) were in tune with each other. Both the programs have predicted that the SNP observed in *mce1A *gene is having the highest pathological relevance. Although, programs used here were primarily designed for predicting functional consequences of nonsynonymous SNPs in human proteins. However, concurrent observations on the nonsynonymous SNPs of *mce *operon proteins reported by both PolyPhen and PMut substantiate our hypothesis further. Energy minimization studies on the structure of Mce1A protein show that Pro359Ser mutation resulted in the loss of α-helical structure in the mutated protein. Analysis of wild and mutated Mce1A protein structures by HB plot indicates that change in hydrogen bonding interaction pattern in the mutant protein lead to conformational changes. Mutation of proline to serine residue in proteins are known to cause structural alterations by the reduction of α-helix content of protein and decreases protein stability and increase its susceptibility to proteolysis by trypsin [25]. Yazyu *et al. *[26] observed that Pro122Ser mutation could bring about the alteration in the pH of the system by changing the cation specificity of melibose carrier (a membrane bound protein which mediates co transport of α-galactosides with monovalent cations) in *E. coli*. Pro122Ser mutant lost the ability to utilize H^+ ^and made the carrier favorable for Li^+^- melibose co-transport. Serine being a hard Lewis base interacts with hard Lewis acids such as Li^+ ^instead of H^+ ^[26]. Mce1A protein is a cell surface protein [27] so it may be speculated that the aforementioned changes due to Pro359Ser mutation may have a diminishing effect on the stability of protein and thus on the biological function of it.

In a further analysis, we compared the SNPs in the genes of *mce1 *and *mce4 *operons in 59 drug resistant (DR) and 22 drug sensitive (DS) clinical isolates. The comparison of SNPs in the *mce *genes in DR and DS clinical isolates revealed that both *mce1 *and *mce4 *operon genes of DS clinical isolates were more polymorphic than DR clinical isolates. It is possible that while drug resistance provides extra edge to DR isolates, the DS isolates try to enhance their virulence mechanisms and adaptability to hostile intracellular environment by undergoing mutations in them. This is also supported by a report by Shimono *et al. *[28] where they have demonstrated that, unlike wild type *M. tuberculosis*, a strain of *M. tuberculosis *with disrupted *mce1 *operon become hypervirulent.

Further study of larger number of single and multi drug resistant isolates may give a conclusive answer to the significance of such an observation.

Taken together the SNP analysis and *in silico *modeling reported here predict that the SNPs in the *mce1 *and *mce4 *operons in the clinical isolates are reasonably frequent. Also, the *in silico *modeling of nonsynonymous SNP in the *mce1A *gene of *mce1 *operon indicates that such change may translate into altered function of the gene that may reflect on the virulence and biology of the pathogen.

## Conclusions

In the present study, we have investigated the extent of polymorphism in the genes of *mce1 *and *mce4 *operons of *M. tuberculosis *in a panel of four standard reference strains (H37Rv, H37Ra, LVS (Low virulent Strain) and BCG) and 112 clinical isolates. Our results show that SNPs in the coding sequences of *mce1 *and *mce4 *operons in clinical isolates can be significantly high. Twenty SNPs were discovered in the two operons out of which 12 were nonsynonymous changes. Further analysis of pathological relevance of these changes revealed that five of the SNPs were deleterious. Overall, *mce4 *operon is significantly more polymorphic than *mce1 *operon (p < 0.001). However, nonsynonymous SNPs detected in *mce1A *gene of *mce1 *operon predict effect of such SNPs on the biology of the pathogen.

## Methods

### Bacterial Strains

A collection of ~112 *M. tuberculosis *clinical isolates and four standard refrence strains (H37Rv, H37Ra, LVS (Low virulent Strain) and BCG) were taken for the study. These isolates were from the patients visiting the out patient department (OPD) of Vallabhbhai Patel Chest Institute, Delhi, India. The strains were collected from sputum samples submitted to the Department of Microbiology for laboratory diagnosis of tuberculosis. The study was approved by the institutional ethics committee. Informed consent was also signed by the patients included in the study.

### Processing of the sample

The sputum samples were decontaminated by the standard Petroff's method [29] and inoculated on Lowenstein Jensen (LJ) media. DNA was extracted from the cultures by the CTAB method [30]

### Drug Susceptibility assays

Drug susceptibility testing was performed by the proportion method. The drug concentrations tested as per WHO recommendations were 0.2 mg/litre for isoniazid, 40 mg/litre for rifampicin, 2 mg/litre for ethambutol and 4 mg/litre for streptomycin. The drug incorporated LJ slants were incubated at 37°C and observed at 28 and 42 days of incubation [31]. The drug susceptibility was carried out on 59 DR and in 22 DS isolates out of the 100 clinical isolates and in 12 random selected isolates.

### PCR amplication

Sixteen genes of *mce1 *and *mce4 *operons were amplified using overlapping primers listed in Additional file 1 for 4 standard refrence strains (H37Rv, H37Ra, LVS (Low Virulent Strain) and BCG) and 12 clinical isolates. Thermal cycling was carried out for 40 cycles, with initial denaturation at 95°C for 10 minutes, followed by denaturation at 94°C for 1 minute, annealing at 56°C-64°C for 1 minute depending on primer sequence, elongation at 72°C for 1 minute and a final extension of 72°C for 10 minutes. The amplicons were purified by Qiagen PCR purification kit to remove unincorporated nucleotides and dNTPs.

### Sequencing and Data Analysis

The PCR products obtained by using the overlapping primer sets as described above from four standard reference strains (H37Rv, H37Ra, LVS (Low Virulent Strain) and BCG) and 12 clinical isolates were sequenced using a DNA sequencer 3730 (Applied Biosystems). Both strands were sequenced to confirm the sequence data. The sequences were aligned and compared with the reference sequence of H37Rv available in public domain at Tuberculist http://genolist.pasteur.fr/TubercuList/[11] using Align two sequences (bl2seq) of BLAST http://blast.ncbi.nlm.nih.gov/Blast.cgi[32]. The SNPs obtained by the sequence analysis were used to screen other 100 clinical isolates through Sequenom MassARRAY system. All the SNPs were analysed further for the change in amino acids in the corresponding protein sequences through Gene Runner software version 3.05 (Hastings Software, Inc.) available at http://www.generunner.net.

## Computational methods

### Structure homology-based method (PolyPhen) to predict functional and structural changes in proteins

In order to analyze the impact of nonsynonymous SNPs on the structure and function of proteins of *mce *operons, Polyphen server http://genetics.bwh.harvard.edu/pph/[33] was used. Protein sequences in FASTA format with the position of amino acid variants indicated were submitted as the query. Polyphen server calculates position- specific independent counts (PSIC) scores for each of the two variants based on the parameters such as sequence-based characterization of the substitution site, profile analysis of homologous sequences, and mapping of the substitution site to a known protein's three dimensional structure and then the difference between the PSIC scores of the two variants are computed. The higher the PSIC score (> 1.5) difference, the higher the functional impact a particular amino acid substitution is likely to have.

### Neural network-based sequence information method (PMut) to predict pathological character of nonsynonymous SNPs

PMut server http://mmb2.pcb.ub.es:8080/PMut/[34] was used to predict pathological relevance of nonsynonymous SNPs in the *mce *operon proteins. The software uses different kinds of sequence information to label mutations from the databases of disease-associated mutations (DAMU), and neural networks (NNs) to process the databases of DAMUs and neutral mutations (NEMUs). The resulting vector of properties is then utilized to decide whether the mutation is pathological or not. Although, PMut is designed to analyze pathological character associated with mutations in the human proteins. A number of workers [35,36] have qualitatively interpreted the functionality of mutated non-human proteins especially that of microbes. We submitted the protein sequences as the query, the location of the mutation and the amino acid residues were also furnished. Small NN (20 nodes, 1 hidden layer) with using 2/3 input parameters (pam40 matrix index, pssm index, variability index) was used to train the database as it is recommended for predictions of non-human proteins [34]. NN output greater than 0.5 is predicted as pathological otherwise neutral.

### Molecular Modeling of nonsynonymous SNP located on the protein structure

The homology modeled three dimensional structure of Mce1A protein submitted to protein databank [PDB: 1NA9] [16] was used to study the effect of mutated residue. The authors have modelled Mce1A structure from residues 68 to 376, the N-terminal 67 residues and the C-terminal 78 residues were not modelled due to lack of homology [16]. Biopolymer module implemented in InsightII (Accelrys Inc.: San Diego, CA) was used to modify the mutated residues, from the InsightII fragment library. Using the same module, hydrogen atoms were added to both wild type and mutated protein structures at pH 7.0. The default cvff (Consistent Valence Force Field) force field [37] was applied to both the structures. Further, a series of energy minimization steps were performed on both the protein structures by InsightII/Discover (Accelrys Inc., San Diego, CA) using the following protocol: (a) In the first step of minimization, all the heavy (all non-hydrogen) atoms were constrained, the hydrogen atoms were allowed to minimize by steepest decent algorithm until the maximum derivative (|dE/dr|) of the system was <1 kcal/(mole.Ǻ). (b) This step was followed by another steepest descent minimization with the same parameter as in step (a), but constraining the protein backbone atoms and relaxing all other atoms of the molecule. (c) In the final step, the protein molecule was minimized by conjugate gradient method with the backbone atom fixed and allowing all other atoms relax until the maximum derivative was <0.01 kcal/(mole.Ǻ). The deviation between the two structures is evaluated by their RMSD values which could affect stability and functional activity. Structure analysis of protein after energy minimization of protein structure was analyzed using Discovery Studio 2.5 (DS Modeling 2.5, Accelrys Inc.: San Diego, CA).

### Statistical methods

Statistical analysis was done by Fischer's exact t test using Graph Pad Prism software http://www.graphpad.com/quickcalcs/contingency1.cfm. A two-tailed p-value < 0.05 was considered statistically significant.

## Authors' contributions

MB and VB conceived the study. MVB provided the clinical isolates of *Mycobacterium tuberculosis*. RP carried out the major experimental work. MC and PP conducted the computational work. AC and NKS helped in experimental design. MB, VB, MVB, RP and PP participated in data interpretation and manuscript preparation. All authors read and approved the manuscript.

## Supplementary Material

Additional file 1 **Overlapping primers to sequence entire *mce1 *and *mce4 *operons**. Click here for file
