# Supplementary material for: Single nucleotide polymorphism in the genes of mce1 and mce4 operons of Mycobacterium tuberculosis: analysis of clinical isolates and standard reference strains
Source: BMC Microbiol. 2011 Feb 23;11:41. doi: 10.1186/1471-2180-11-41 (PMC3050694; doi:10.1186/1471-2180-11-41)
Supplement: Additional file 1 — Overlapping primers to sequence entire mce1 and mce4 operons. [file 1471-2180-11-41-S1.DOC]

**Additonal file 1-Overlapping primers to sequence entire *mce1* and *mce4* operons**

| **Primers** | | | | | |
| --- | --- | --- | --- | --- | --- |
| ***mce1* operon** | | | ***mce4* operon** | | |
| **Primer Name** | **Forward Primer** | **Reverse Primer** | **Primer Name** | **Forward Primer** | **Reverse Primer** |
| A1 | TAACGTTTGCCCGCTATGAG | GGGCCAGCAGAATATTGAGC | 4.1 | CGATCTGATGCGTCAGTG | ACGCAACAGAATGTTGAG |
| A2 | CTGCAGTGCTGGTTCATCAC | ACCGATGGTCGTCAAGATCG | 4.2 | GACGTTGCTGGTGTCCATTC | GATCTCCGCCAGTATCAATTG |
| A3 | CGTGATCTTGACGACCATCG | AGGGCCTCCAAGAAGGACC | 4.3 | TGCTG TCAACGGCTTGGTG | TGGGCACATAGCGCATGGTTC |
| AJ | ATTATGTCGTTCCTGTCCCC | GGTGAGCGTCTGGAACAAC | 4.4 | CTGTTCTACGGCGAAACATG | CAGCACCGACAACGAATACC |
| A4 | GGGAGACAACAAGGAGATC | GATAACGCCAAAAGACG | 4.5 | ACTCGTGTCCTATTTGG | GGACATGTTTCCTTTCTGAAG |
| A5 | CTGAGGACGAACTCAGAGC | GATATTGAACTCCACCCGAC | 4.6 | GTAACTTCAACCTCTCGG | CTCAAGCTGTACCTGAGAC |
| A6 | GCAAGGTCAAAGCACTACAC | TTCTGGAAATCGCCGTAGG | 4.7 | TTCATTCCGCCAAAGACG | GGCGTCGATGAAATTCTG |
| A7 | TACCTGGACGCTATTCAGC | TCGGAGAACTTAGCCACC | 4.8 | ACCTCAACGACACGCTGCTAG | ACGGCGCGTCAACCTGTAAC |
| A8 | ACATCGAGACGGTCAAGC | TGAGTGAGGTTAGCGCAGG | 4.9 | ATATCCCGACCAAGCAGAC | ATGCTGTGGTCTGGGTTG |
| A9 | TGCGCTAACCTCACTCAAGG | ATTATCGGCCAGCACTGG | 4.10 | TACCCGCCGATCATGTTCC | CCACATCAGCCATTTCGAC |
| A10 | GAACAAGTACAAGGTGCCC | GTCTCCAACCCATCCAACG | 4.11 | AGTTTGGCGGGCTGAACTC | GGGTCTCGTCAATGATCTCTTC |
| A11 | GTGACCACCACGTTGCTGC | ATCATTGACCCCCTCCTGC | 4.12 | GGTCAACAAGGGTAACGTC | AAG TTGGGAAATGGGAAC |
| A12 | GGTTTACAGGCCCCACAGG | GCGCGGGTAATGTCATCG | 4.13 | CGAGACCAAGGTGGATT CGG | AGATCGCCATCACGCTCAGG |
| A13 | TTCACCGACGAGCTCAACC | TGTACTGCCCGATACCCACC | 4.14 | AAGGCAGGCCAGAAGGTTC | GCGTTGAGGTTGGTGATTAC |
| A14 | TGCTGACTCGCTTCATCC | TGATCACCCGTGTTGACC | 4.15 | ACCAGTGCGGTCATCGAGTTGC | CTTGATCGTCACCGAGCAGAAG |
| A15 | TGGACAGCCAGGTCAACAC | TTTGCCCAGGACATCGAC | 4.16 | GCAGTTACGGAGCATTCTTC | AATCGTGGTCGACTTGCC |
| A16 | AACATTCCCTGTGTGATG | CTCCGTAGACGACGACTTATTC | 4.17 | TGCAAAGGTCACCTTCAGCG | TGCAACATCGTCGATCCCAGAG |
|  |  |  | 4.18 | CTGGGATCGACGATGTTGC | ATAGAGCCCGACAGCAGAG |
|  |  |  | 4.19 | ATCGTGGGCTACTTCACCTCTG | TTGACGGTGCCGAAGATGTC |
|  |  |  | 4.20 | GGAATGGGACGAGGTGAAAGAG | AGATGAACTGCACCGGGTTAGC |
|  |  |  | 4.21 | GAGCGAGAACATCGAGCAAGTG | GCTGCTGGCCGTCAATATG |
|  |  |  | 4.22 | CGCACATGGACGAGATCCTC | ACTTGGTCGACGACGGGTTCTC |
|  |  |  | 4.23 | GCCATCGGTGAGCAGTACATC | CGGGAAACGAAGGACGAATC |
|  |  |  | 4.24 | CCGGGATTCGTCCTTCGTTTCC | TGTTGGGCGGCAGAATGTTTCG |
|  |  |  | 4.25 | GGACATCAAGTCACTCGC | AATTCCTGGCACGGGTAG |
|  |  |  | 4.26 | GCGCCAAGCTGGACTTCAAG | CATGACGTGAACGGTGGAGG |
|  |  |  | 4.27 | GCTATGTGCCAGTTGGAACC | GCATGAGATCCACCCAATTCTC |
|  |  |  | 4.28 | ACCAACTCAGCGGAGCCTTC | GCATAACCAGTGCGGTCACAC |
